# Patients' satisfaction with inpatient services provided in hospitals affiliated to Tehran University of Medical Sciences, Iran, during 2011-2013

**Published:** 2016-06-11

**Authors:** Jalil Makarem, Bagher Larijani, Kobra Joodaki, Sahar Ghaderi, Fatemeh Nayeri, Masoud Mohammadpoor

**Affiliations:** 1 Assistant Professor, Department of Anaesthesiology, Imam Khomeini Complex, Tehran University of Medical Sciences, Tehran, Iran;; 2 Professor, Medical Ethics and History of Medicine Research Center, Tehran University of Medical Sciences, Tehran, Iran;; 3 PhD Candidate in Medical Ethics, Medical Ethics and History of Medicine Research Center, Tehran University of Medical Sciences, Tehran, Iran;; 4 Researcher, Imam Khomeini Complex, Tehran University of Medical Sciences, Tehran, Iran;; 5 Associate Professor, Family Health Institute, Maternal-Fetal & Neonatal Research Center, Tehran University of Medical Sciences, Tehran, Iran;; 6 Assistant Professor, Department of Pediatrics, Children’s Medical Center, Tehran University of Medical Sciences, Tehran, Iran.

**Keywords:** *Patient satisfaction*, *Inpatient care*, *Hospital service quality*

## Abstract

Implementation of patient feedback is considered as a critical part of effective and efficient management in developed countries. The main objectives of this study were to assess patient satisfaction with the services provided in hospitals affiliated to Tehran University of Medical Sciences, Iran, identify areas of patient dissatisfaction, and find ways to improve patient satisfaction with hospital services.

This cross-sectional study was conducted in 3 phases. After 2 initial preparation phases, the valid instrument was applied through telephone interviews with 21476 participants from 26 hospitals during August, 2011 to February, 2013.Using the Satisfaction Survey tool, information of patient's demographic characteristics were collected and patient satisfaction with 15 areas of hospital services and the intent to return the same hospitals were assessed.

The mean score of overall satisfaction with hospital services was 16.86 ± 2.72 out of 20. It was found that 58% of participants were highly satisfied with the services provided. Comparison of mean scores showed physician and medical services (17.75 ± 4.02), laboratory and radiology services (17.67 ± 3.66), and privacy and religious issues (17.55 ± 4.32) had the highest satisfaction. The patients were the most dissatisfied with the food services (15.50 ± 5.54). It was also found that 83.7% of the participants intended to return to the same hospital in case of need, which supported the measured satisfaction level.

Patient satisfaction in hospitals affiliated to Tehran University of Medical Sciences was high. It seems that the present study, with its large sample size, has sufficient reliability to express the patient satisfaction status. Moreover, appropriate measures should be taken in some areas (food, cost, and etc.) to increase patient satisfaction.

## Introduction

The new health care system in the world is moving from a set of purely provider-based systems to receiver-based systems. In this regard, patients' satisfaction is an essential component of quality assessment (1). Recognizing opinions of users and service recipients is a quick and inexpensive way to determine which parts of the service require improvements in terms of quality (2). In addition, the results of these studies may aid insurance payers, regulatory institutions, and validation agencies, as well as hospital authorities and consumers (3).

Various definitions around patients’ satisfaction have been proposed during the last 30 years. Moreover, the basis of these definitions dates back to theories introduced in 1980, in which Investigators proposed 5 broad perspectives on satisfaction (4).

These perspectives include the discrepancy and transgression theories (1981), expectancy-value theory (1982), determinants and components theory (1983) multiple models theory (1983), and healthcare quality theory (1980) (4).

In summary, satisfaction is a complex concept that may be linked to various factors social values (5). Satisfaction can also be described as a subjective concept that is assessed differently by different people. People may even develop different definitions of satisfaction at different times (6).

A review conducted in 2001 postulated that most studies conducted on satisfaction have focused on the 5 axes of nurses, physicians, food, services, and care. Most popular instruments used in this study can be named as adapted SERVQUAL, the Press Ganey Associates instrument and Picker questionnaires (3).

There are various methods for assessing patients' satisfaction including using electronic forms, phone calls, and face to face interviews with patients (3, 7). Performing the Satisfaction Survey using a questionnaire has the features of posing more questions, reducing the possibility of bias, and also cost-effectiveness. Although, sending and receiving questionnaires and low return rates are thought as the major problems of this approach (3).

Telephone follow-up of discharged patients has been proposed in many articles as an accessible and low cost method (7-9) in which a lower time delay is reported compared to other methods (3). Other benefits of post-discharge telephone follow-up are opportunities for direct communication and exchange of information with patients, provision of education and explanation to patients if needed, early detection of complications in patients and management of symptoms and problems, assuring patients, improving the quality of health services, and increasing patient satisfaction and awareness of patients' opinions about the health care delivery system (8, 10).

In many countries, through special attention to the post-discharge period and precise planning, service delivery not only proceeds after discharge, but also patient's treatment and recovery are well monitored by interacting closely with patients. Moreover, the patients are questioned about the quality of medical, nursing, laboratory, preclinical, and accommodation and food services. This aids healthcare providers in improving and enhancing service delivery to patients (11). Surveying patient satisfaction is also helpful in the identification of strengths and weaknesses of each hospital and creates an atmosphere of constructive competition among health care providers in hospitals to enhance the quality of services (12, 13).

Very little is known about the Iranian patients' satisfaction. Moreover, lack of comprehensive studies and continuous assessment in this field has led to many problems. Thus, despite the efforts of health care providers, they are still unable to take advantage of patients' opinions in order to improve service quality. In recent years, several studies have been conducted to assess patient satisfaction; however, they were cross-sectional studies, which frequently suffer from sample size limitations, and/or correlation study (14-19). This study is the first continuous research assessing satisfaction with a valid and large sample size and has responded well to the needs in this area in order to manage the health system in Iran.

In this study, using post-discharge telephone follow-up, patients were asked to express their opinions about the health services available to them. Subsequently, the causes of discomfort were identified and feedback on the strengths and weaknesses of different services were reported to hospital management. Based on these outcomes, improving the quality of medical care in university hospitals were targeted.

## Method

This was a cross-sectional study carried out in 2011-2013. The target population consisted of patients discharged from 26 hospitals affiliated to Tehran University of Medical Sciences (TUMS), Tehran, Iran. This study was performed during the merge of Tehran University of Medical Sciences and Iran University of Medical Sciences; therefore all the twenty six hospitals were affiliated to TUMS.

The study was designed in the form of 3 phases. Permission to carry out the study was obtained from the Research Committee of Tehran University of Medical Sciences with the project code of 91-01-66--16650. In the first phase, described as launching, a valid and reliable researcher-made questionnaire was used for data gathering. The preliminary technical preparations for the study included the collection of initial information such as statistics of admissions and discharges from affiliated hospitals, and recruitment and training of project partners for telephone interviews with patients.

The initial data gathering form was well studied and reviewed by 10 faculty members who specialized in the field of quality assessment and patient satisfaction. Then, the required modifications and necessary changes were made and content and formal validity of the instrument was approved by an experts group.

The second phase, pilot implementation, was implemented with the following procedures. After coordination with authorities of 3 university affiliated hospitals, the project was performed in the form of 2 pilot studies on 30 patients (to assess construct validity) and on 300 patients (to identify and resolve study implementation problems). Accordingly, the necessary changes in the data collection form and method were applied. After re-examining, Cronbach's alpha of 0.87 was calculated for the present questionnaire (α = 0.87). 

In the third phase, final implementation, the project was implemented on a consistent basis in all the hospitals affiliated to Tehran University of Medical Sciences.

In this study, we examined all patients with a history of hospitalization in hospitals affiliated to Tehran University of Medical Sciences in the timeframe of August, 2011 to February, 2013. The studied hospitals included Arash, Imam Khomeini, Shariati, Baharlou, Rasoul Akram, Akbarabadi, Ali Asghar, Amir A'lam, Bahrami, Farabi, Firouzgar, Hasheminejad, Mirza Kuchak Khan, Hazrat Fatemeh, Rouzbeh, Sina, Shahid Motahari, Razi, Vali-e-Asr, Shafa Yahyaeian, Ziaeian, and Shahid Rajaee Hospitals, and Tehran Psychiatric Institute, Cancer Institute, Children's Medical Centre, and Tehran Heart Center. Note that patients hospitalized in emergency wards were excluded from our study. 

In the present study, 10% of the study population were selected using a systematic sampling method, and they were telephoned during the first 3 days after discharge from the aforementioned healthcare units. 

The Satisfaction Survey form used in this study consisted of 3 main parts. The first part included patients' demographic characteristics such as age, gender, place of residence, and relation of responding person to the patient. The second part consisted of 5 variables related to hospitalization (name of the admitting hospital and ward, length of hospitalization). The third part extracted answers to 15 questions which assessed different aspects of patient satisfaction with hospitals. Patients rated their satisfaction level with a number between 0 and 20. This rating system was used due to the ease of understanding grading between 0 and 20 as individual respondents.

Patients' satisfaction was determined in terms of satisfaction scope in the domains of physicians, nurses, laboratory and radiology services, ancillary staff services, administrative staff, guardians, clarification of the disease by medical personnel, getting well after discharge, food, administrative procedures before admission, hospital accommodation, hospital sanitary procedures, compliance with legal and ethical issues and patient's religious boundaries, availability of medical facilities, discharge administrative procedures, and hospital costs. 

The participants were asked in regards to their reason for choosing the hospital for treatment, and the intent to return to the same hospital in the case of need and the main reasons for this intent. In addition, the effect of cost-effectiveness in hospital choice was reviewed.

An average of 15 minutes (at least 10 minutes) was dedicated to each interview for every person. At the beginning of each interview, the operator introduced herself to the participants, explained the objectives of the project, and obtained a verbal consent of participation to continue the study. In case of patient’s reluctance to cooperate in the study, another participant discharged from the same hospital was replaced using random sampling. During this period, a total of 22727 patients were contacted. Of these, 96.1% were willing to participate in the study. Descriptive analysis of the data was carried out using SPSS software (SPSS Inc., Chicago, IL, USA) and Student’s T-test, ANOVA, and the Pearson correlation.

## Results

Twenty one thousand four hundred seventy six patients participated in this study. The distribution of participants in different hospitals is shown in table 1.

**Table1:** Number of participants according to hospitals

Hospital name	Number of participants	Hospital name	Number of participants	Hospital name	Number of participants
**Razi**	186	Hazrat Fatemeh	321	Akbar Abadi	1870
**Rouzbeh**	102	Tehran Heart Center	1051	Ali Asghar	349
**Shariati**	1640	Mirza Kuchak Khan	422	Amir A'lam	989
**Sina**	843	Shahid Motahari	134	Arash	859
**Children's Medical Centre**	1249	Shahid Rajaee	1058	Baharlou	737
**Vali-e-Asr**	1421	Rasoul Akram	1262	Bahrami	602
**Shafa Yahyaeian**	563	Iran Psychiatric Institute	76	Cancer Institute	1080
**Hasheminejad**	518	Firouzgar	746	Ziaeian	622
**Imam Khomeini**	1622	Farabi	1154	***Total***	*21476*

Distribution of age, gender, and other variables of participants were assessed (Table 2). The correlations between patients' satisfaction and their demographic characteristics were studied and the existence of significant differences was assessed, the most important of which are presented in table 2.

**Table 2:** Distribution of demographic characteristics and significant differences/ correlation between/with overall satisfaction

Demographic characteristics	**Frequency**	**Percentage**	**Satisfaction score ** **(mean ± SD)**	**Results of significant tests**
Gender ***Male*** ***Female***	9436 11981	44 54	16.66 ± 2.83 16.86 ± 2.70	* ---
Age (year) *** > 20 *** ***20-49*** ***50-69*** *** < 70***	4906 10279 4258 1572	23.3 48.9 20.3 7.5	17.06 ± 2.44 16.67 ± 2.84 16.77 ± 2.78 16.55 ± 3.04	Spearman (C coefficient) = -0.029 (*P* = 0.001)
Residency ***Tehran*** ***Other cities***	13009 8283	61.1 38.9	16.78 ± 2.75 16.99 ± 2.67	(T-test for equality of variance) T = -5.36 (*P* = 0.001)
Respondent ***Patient*** ***Relatives***	11628 9987	51 49	16.87 ± 2.70 16.65 ± 2.83	(t-test for equality of variance) T = 5.77 (*P* = 0.001)
The length of stay in hospital				Median: 3 days Range: 1-191 days

* *no significant differences between gender-based satisfaction scores*

The mean score of participants' satisfaction in all areas of hospital services was 16.86 ± 2.72 out of 20. Accordingly, overall satisfaction rate of 58.4% of participants was higher than 17 and was lower than 10 in only 2.8% (Table 3).

As shown in table 4, patient satisfaction was assessed in each service area. Among the surveyed areas, the physician domain had the highest satisfaction score. In the second place was the area of laboratory and radiology services, and next was compliance with legal and ethical issues. The lowest satisfaction score was reported in the area of hospital food. Furthermore, the hospital accommodation and paid fees, respectively, were among the lowest satisfaction scores.

**Table 3:** The distribution of participants in the satisfaction scores group

Overall satisfaction score	Frequency	Percentage
**< 10** ** (low satisfaction or not satisfied)**	594	2.8
**10-14.99** **(moderate satisfaction)**	3450	16.2
**15-16.99** **(moderate to high satisfaction)**	4812	22.6
**17-20** **(high satisfaction)**	12436	58.4
**Total**	21292	100

**Table 4:** Satisfaction rate in each scope

Service area	Mean score ± SD	The percentage of participants with high satisfaction (score = 17-20)	The percentage of participants with low satisfaction (score < 10)
**Physicians**	17.75 ± 4.02	77.8	7.3
**Laboratory and radiology services**	17.67 ± 3.66	73.6	6.4
**Legal and ethical issues**	17.55 ± 4.32	76	9.1
**Ancillary staff services**	17.54 ± 3.78	71.7	7.1
**Administrative procedures of discharge**	17.48 ± 3.95	72.2	8
**Availability of medical facilities**	17.45 ± 4.25	73.8	9.4
**Administrative staff**	17.40 ± 3.87	70.2	7.1
**Guardians**	17.38 ± 4.13	70.4	8.1
**Nursing services**	16.82 ± 4.40	65.7	10.6
**Administrative procedures before admission**	16.71 ± 4.64	65.6	12.6
**Hospital sanitary procedures**	16.52 ± 4.75	62	12.2
**Clarification of the disease by medical personnel**	16.43 ± 5.45	66.9	14.9
**Getting well After discharge**	16.42 ± 4.75	62.1	12
**Hospital costs**	16.23 ± 4.82	6.1	15.7
**Hospital accommodation**	16.15 ± 4.85	57.4	14.3
**Food services**	15.50 ± 5.54	54.9	19

**Figure 1:**
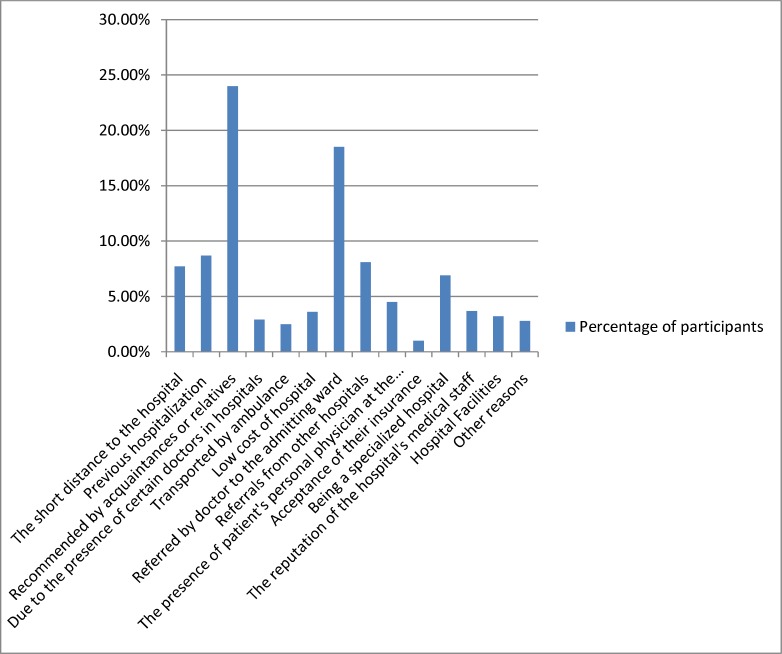
Distribution of participants based on reasons for choosing the hospital

In this study, the most important reasons for choosing the hospital by the participants were explored. These reasons were, respectively, recommended by acquaintances or relatives (24%), referred by doctor to the admitting ward (18.5%), and previous hospitalization (8.7%). The least important factor in hospital choice was declared by patients as acceptance of their insurance (1%) (Figure 1).

**Figure 2:**
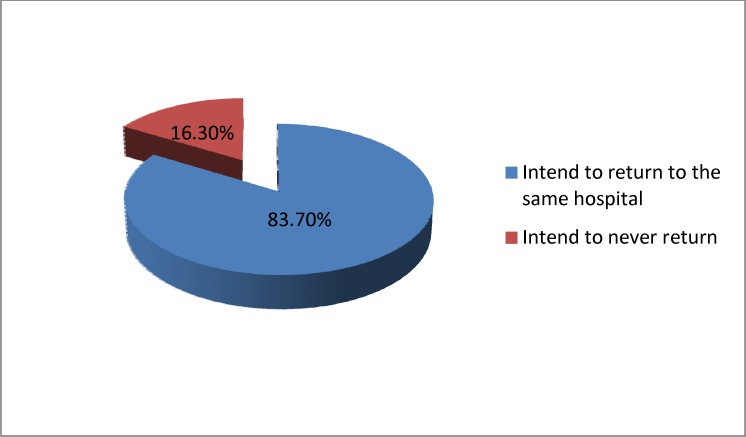
Distribution of participants based on the intention to return to the same hospital if needed

In the present study, 83.7% of the participants stated that in case of relapse and the need for more health care they will choose the same hospital. The most commonly cited reasons for reselection of the same hospital were satisfaction with physicians, overall satisfaction with hospital, and satisfaction with dealing with patients, nursing care, and hospital facilities. However, 16.3% of patients said they will not come back to the same hospital in case of needing future care. The most important reasons noted were dissatisfaction with physicians, interaction with patients, and nursing care, poor sanitary conditions in the hospital, and hospital charges (Figure 2).

**Figure 3:**
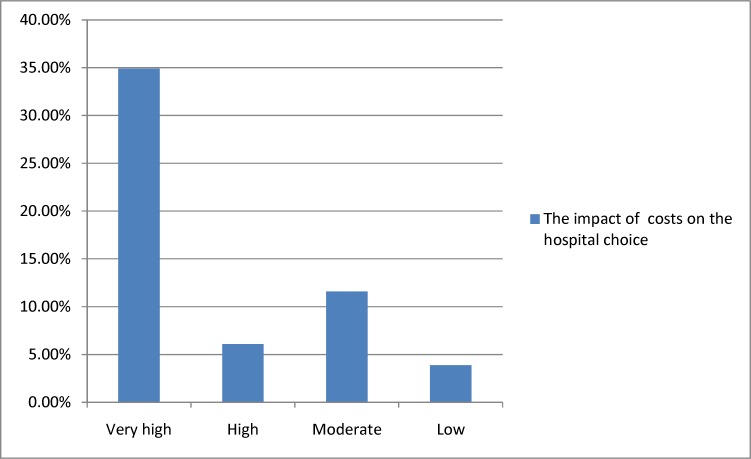
The impact of costs on hospital choice

In figure 3, the impact of cost on the hospital choice by the participants is presented.

***Discussion and Conclusion***

The purpose of this study was to investigate the causes and domains of patients' dissatisfaction with hospitals affiliated to Tehran University of Medical Sciences. The present study was also aimed at providing guidelines to improve hospital services and to increase patient satisfaction. An average satisfaction of 16.86 ± 2.72 with the overall services provided to patients was reported, and 50.8% of participants reported high satisfaction with health care services, which was consistent with results of previous studies (20, 21).

Among the demographic characteristics of the participants, increasing age and duration of hospitalization were associated with lower satisfaction. Participants in the age group of older than 70 years had the lowest levels of satisfaction, which was inconsistent with the results of many studies (22-25). This is justified considering chronic diseases in elderly patients and their lower health level compared to young patients (23, 25, 26). No significant statistical differences were observed in terms of gender, which is consistent with many studies (22-24). Nevertheless, some studies have reported higher satisfaction in women (9, 27), and sometimes in men (23).

In many studies, duration of hospitalization had negatively impacted patients' satisfaction (28, 29). Several studies reported no negative impact of treatment duration and patient satisfaction which can be due to ensuring continuity of treatment and concerns about the lack of sufficient information to continue treatment and care at home (27, 30).

Satisfaction among patients who live in Tehran has been reported as lower than other cities. Overall, patients' relatives have reported lower satisfaction scores in terms of the services. Review of studies have shown that providing clear and consistent communication with physicians and getting answers to questions, and use of spiritual support can be effective in reducing anxiety, and therefore, increasing satisfaction among patients' relatives (31-33).

In several studies, patients' satisfaction had a significant association with their intention of returning to the same hospital or recommending it to others (34, 35). In this study, 83.7% of participants said that in case of future need of medical services they will choose the same hospital, which confirms the degree of satisfaction to services received. This is more or less proportional to the statistics published in previous studies.

In this study, the main reasons of participants for their intention to return were satisfaction with physicians, overall satisfaction with hospital, and satisfaction with interaction with patients, nursing care, and hospital facilities. A study has shown that the only predictor which is significantly associated with patients' intention to return to the same healthcare center is satisfaction with clarifications provided by physicians and expected time for waiting to be visited by physicians (35).

The findings of this study showed that the most common ways of hospital choosing from the perspective of the patients are, respectively, recommendation by others, referred by doctor, and previous hospitalization. These findings are consistent with previous studies claiming that, due to lack of available public information, communication channels such as friends and relatives of the patient are the most influential channels for choosing a hospital (36, 37). Acceptance of insurance had the least impact on participants' choice. This finding can be justified due to acceptance of all types of insurance in hospitals affiliated to Tehran University of Medical Sciences.

The main reason for the high level of patient satisfaction may be satisfaction with performance of clinicians. In previous studies physicians’ performance has been proposed as one of the most important predictors of patients’ satisfaction (14). Physician’s attitude toward the patient has a dramatic impact; this can be detailed as compassion and willingness to provide information and clarification for patients (38). Furthermore, previous studies have noted the importance of healthcare providers' and caregivers' attitudes to be even more than their technical skills (39-42). 

Based on the above findings, identifying approaches to increasing physician empathy and continuous education of communication strategies to clinical staff are recommended in order to improve patient satisfaction.

In the following ranks, the area of radiology and laboratory services in the hospital was assessed as strongly suitable by patients. This finding was in agreement with that of the study by Sanders (43). The most important factors in satisfaction with radiology services were appropriate scheduling and short waiting time (44-46). Improvement of staff interactions plays an important role in increasing patient satisfaction in this domain (45-47). 

Compliance with spiritual issues was the third most important factor shaping patients’ satisfaction. Taking ethical and religious issues into account clearly influences patients’ satisfaction, especially for patients with severe pain, chronic diseases, and life threatening complications (48-50).

In this study, the lowest satisfaction score of hospital services was assigned to the domain of nutrition and accommodation. In a study conducted in Mashhad, it was shown that patients' satisfaction is affected by nutrition as well as the quality of hospital rooms (51).

In contrast, in most studies abroad, patients' satisfaction with hospital food services have been reported as higher than 80%, or have been rated as good or very good (52, 53). This indicates that special attention to nutritional status and accommodation in public hospitals is essential. In addition, periodic surveys of patients to benefit from their opinions about the quality of nutrition and accommodation, while attending to the principles of good and healthy nutrition, are effective and fruitful in improving the healthcare services.

It has been shown that the physical environment of hospitals (hospital services) is effective in patient satisfaction (54, 55). Our suggestions for the improvement of the rate of patient satisfaction are respecting the physical privacy of patients during hospitalization and improving their accommodation requirements. We recommend the improvement of accommodation in terms of sufficient space, lighting, noise reduction, standard health services, and provision of sufficient restrooms and adequate number of personnel to aid patients’ everyday activities, and renewal of old constructions according to international standards and requirements of Iranian patients and medical staff. 

In the present study, the main reasons for patients' dissatisfaction were high hospital charges, lack of health recovery, lack of provision of information about the treatment process and clarification of information for patients.

Due to fundamental differences in insurance regulations and hospital charges between Iran and most advanced countries, comparing of patients' satisfaction of cost-effectiveness is not feasible. Undoubtedly, eliminating direct financial transactions with patients, improving insurance services, extending insurance coverage, and providing free treatment can have a significant impact on patients' satisfaction.

In an article has been concluded that despite the relationship between satisfaction and treatment outcome, when patients are asked to rank their satisfaction of healthcare services, they pay more attention to their health status after treatment rather on improvement of symptoms (56). However, future studies should place more emphasis on early symptoms of improvement rather than treatment outcome.

Many studies have addressed the importance of providing information and education for patients (57), and in many cases, similar to our findings, patients feel unsatisfied with the domain of provision of adequate clarification and information by caregivers (54, 58). The lowest level of satisfaction with hospital staff was assigned to nurses. Note that a clear definition of nursing practices and their specific tasks is not provided for patients. 

Nurses also play very significant roles in patient satisfaction whether in terms of nursing care or overall healthcare services provided to patients (38, 59, 60).

Incorporation of patients’ preferences into treatment decisions by physicians and nurses can enhance their satisfaction (59). Characteristics of a good relationship between care providers (physicians and especially nurses) and patients are common understanding, respect, trust, honesty, and a sense of good humor and amiability (60).

Previous studies have stressed that provision of individual-centered services by nurses and more attention from nurses can enhance the satisfaction level (38, 54, 61). Technical and clinical skills of nurses are also an important factor in patient satisfaction (61). Furthermore, providing adequate information and skills, cooperating with physicians during testing and treatment (54), and especially healing and reducing patients' pain significantly increase satisfaction (62).

In studies carried out in Iran, a variety of factors are proposed as leading to patients' dissatisfaction such as educating patients (15), responding to questions and requests of patients (16-18), and communication skills (19). Therefore, it seems that exploring reasons for low satisfaction with nursing services in hospitals affiliated to Tehran University of Medical Sciences necessities more detailed and specific investigations.

Moreover, in continuation of this study, there is a need to integrate more precise studies to explore different aspects of dissatisfaction in various domains and to explore various associated factors. In addition, using specialized questionnaires in specific populations, such as psychiatric patients, is essential to obtain more reliable data.

It seems that, due to the large sample size of this study, it possesses enough reliability to assess the status of satisfaction among patients. Although in some areas (nutrition, accommodations, and etcetera), It is essential that, as soon as possible, the necessary steps be taken to enhance satisfaction with healthcare provisions.
